# Serial Deaths of Young Trainee Physicians in Iran During COVID-19 Pandemic; Messages to Policy Makers

**DOI:** 10.3389/frhs.2022.777065

**Published:** 2022-05-13

**Authors:** Reza Gharebaghi, Fatemeh Heidary, Ali Asghar Pourezzat

**Affiliations:** ^1^International Virtual Ophthalmic Research Center (IVORC), Austin, TX, United States; ^2^Department of Public Administration, Faculty of Management and Faculty of Governance, University of Tehran, Tehran, Iran

**Keywords:** physician residents, mortality, Iran, COVID-19, healthcare worker, fellowship, brain drain, migration

## Introduction

The untimely and serial death of the physician residents in recent months in Iran shocked the public and the profession, and refocused consideration on young physicians.

Physician residents refers to early career physicians in training immediately after graduation from medical school (1). Although the exact reason for this mortality of physician residents is not yet known, social activists believe that the frustrating long shift hours, sometimes involving working in the hospital for 72 h with no break, the educational conditions, difficult jobs, limited facilities, and negligence of authorities are responsible for this disaster.

This story of extreme work pressure and lack of support, which has been experienced as unbearable, is pure tragedy (2). This paper presents evidences with recommendations to policy makers on this issue.

### Break the Silence: Angels Are Dying

There is no official report on physician trainee's death in Iran; however, local reports have shown that the 13th physician trainee in the past year has lost his life (2). Generally, physicians constitute a profession at-risk of suicide. However, the rate of suicide has also been reported to be increasing in trainees (3). Therefore, it is suggested that healthcare workers, including physician trainees, should have access to mental health consultation and early detection and treatment of potential illnesses including anxiety disorders and depression, as well as self-help resources along with the determination of a cause of this pathology.

### Immigration and Shortage of Healthcare Workers

The World Health Organization (WHO) estimates that the world may face a global shortage of almost 4.3 million healthcare workers. The crisis in the healthcare human resource has been labeled as one of the most pressing global health challenges (4). To date, 3,000 physicians have lodged emigration applications at the Medical Council of Iran (5) and 300 healthcare workers in Iran have died of COVID-19 within 18 months (6). On the other hand, many subspecialty fields are vacant, with reports showing the collective withdrawal of young physicians from anesthesiology, emergency medicine, internal medicine, and infectious disease departments (7). These unfortunate facts require swift and appropriate intervention. While promises have been made to improve the situation, the lack of policy interventions risks the healthcare human resource crisis to become severe and consequently to limit the availability of health services.

### A Big List of Challenges

Table 1 summarized a list of challenges before and after graduation that physician trainees in Iran may have (2, 3, 7). This is a serious situation that demands urgent attention. Medical residents pass a difficult examination and submit an affidavit to practice in disadvantaged regions in order to be admitted to the residency training program. This commitment must be upheld even after the death of the affiant, when his/her family are obligated to pay compensation to the government when the deceased cannot meet their obligation. These young physicians are mostly in the prime of their life, in the range of 25–30 years of age. They are not civil servants, and are paid a trivial amount, around 100 United States Dollar per month, while they are providing a full-time service, which is even less than the pay of a construction worker or even a street sweeper. Such a salary typically does not even cover rental accommodation. They work long shifts, sometimes up to 400–500 h per month, while an unskilled construction worker works about one-third of these hours for almost twice the wage. In Iran, the residency program takes 3–5 years to finish. Residents work for longer hours and receive lower salaries than any other job. Moreover, most of the time, they have no supporting system, such as liability insurance, retirement fund, or social security insurance. At the same time, they are not allowed to practice in the private sector for their free time outside the hospital (Table 1).

**Table 1:** List of challenges that trainee physicians may encounter in the health system in Iran.

Challenges prior to graduation	➢ Low salary, bonus, profit-sharing, and no overtime pay
	➢ High number of night shifts, particularly for first-year trainees
	➢ Lack of full employee benefits
	➢ Unsuitable on-call rooms in hospitals
	➢ Insufficient personal loans for emergencies
	➢ Impossibility of working in the private sector while studying
	➢ Lack of medical and dental insurance for residents and their family members
	➢ Lack of social security insurance, social support, and communication, leading to maladaptive coping mechanisms
	➢ Lack of accommodation facilities after shifts (e.g., university campus)
	➢ Concerns about proper personal protective equipment and booster dose of COVID-19 vaccine
	➢ Long period of self-isolation away from close relatives during COVID-19 pandemic
	➢ No maximum number of shifts and working hours
	➢ Insufficient opportunities for study and research
	➢ Non-observance of professional ethics by superiors
	➢ Inability to take full advantages of leave while studying
	➢ No direct channel to report burnout issues or present whistleblower reports
	➢ Compelled to publish dissertation outcomes in an indexed journal as a graduate requirement
	➢ Lack of supervision system on annual examination appeals and remark request
	➢ Lack of clinical mentoring programs
	➢ Extra-legal councils and approvals committees (e.g., 7-member council)
	➢ Disagreement of education deputy to decisions of Court of Administrative Justice
	➢ Lack of complete trust to the annual departmental evaluations
	➢ Insistence of Health Ministry to complete examinations even at the peak of the COVID-19 pandemic
Challenges after graduation	➢ Lack of full employee benefits
	➢ Low salary, bonus, and profit-sharing
	➢ Unjust tax cut in comparison to other jobs
	➢ Lack of transport and accommodation facilities
	➢ Irregular payment of physician's salary
	➢ Distance from the spouse's place of study and work
	➢ Long-term commitment to serve the government
	➢ Lack of timely counseling services and social and mental support
	➢ Low health care services tariffs in Iran
	➢ Restriction on private entrepreneurship

Fatigue, frustration, heavy responsibility in medical wards, undeserving treatment from higher levels, assignment of increasing numbers of shifts as punishment, and witnessing the untimely death of their colleagues have given this group a sense of abandonment (Table 1). They have a sense of being completely neglected and unseen, which in turn could result in unintentional errors and a decrease in the quality of medical services.

The reports show that new physicians are not being recruited; instead, residents are assigned to work in COVID-19 wards. There have also been instances where specialists in unrelated fields, such as ophthalmology, have been used to triage cases in COVID-19 sectors.

Researchers believe that fostering healthcare working conditions that supports intrinsic motivation and improves working hours, as well as rewarding physicians fairly and equitably may preclude burnout and job dissatisfaction (8).

In Iran, medical trainees, who are mostly of marriage or reproductive age, either show no intention to start a family due to high work pressure and stress; or, if they marry, do not have children because of their poor economic conditions and considering that childbirth-related leave is not included in their service period.

Although medical education in Iran is free, those who are transferred from foreign universities to state universities are forced to take a supplementary course and pay a heavy annual tuition fee to the universities. These individuals sometimes do not even receive a small salary. The main challenges of physician trainees are listed as below.

#### Financial Struggle

**Figure 1:**
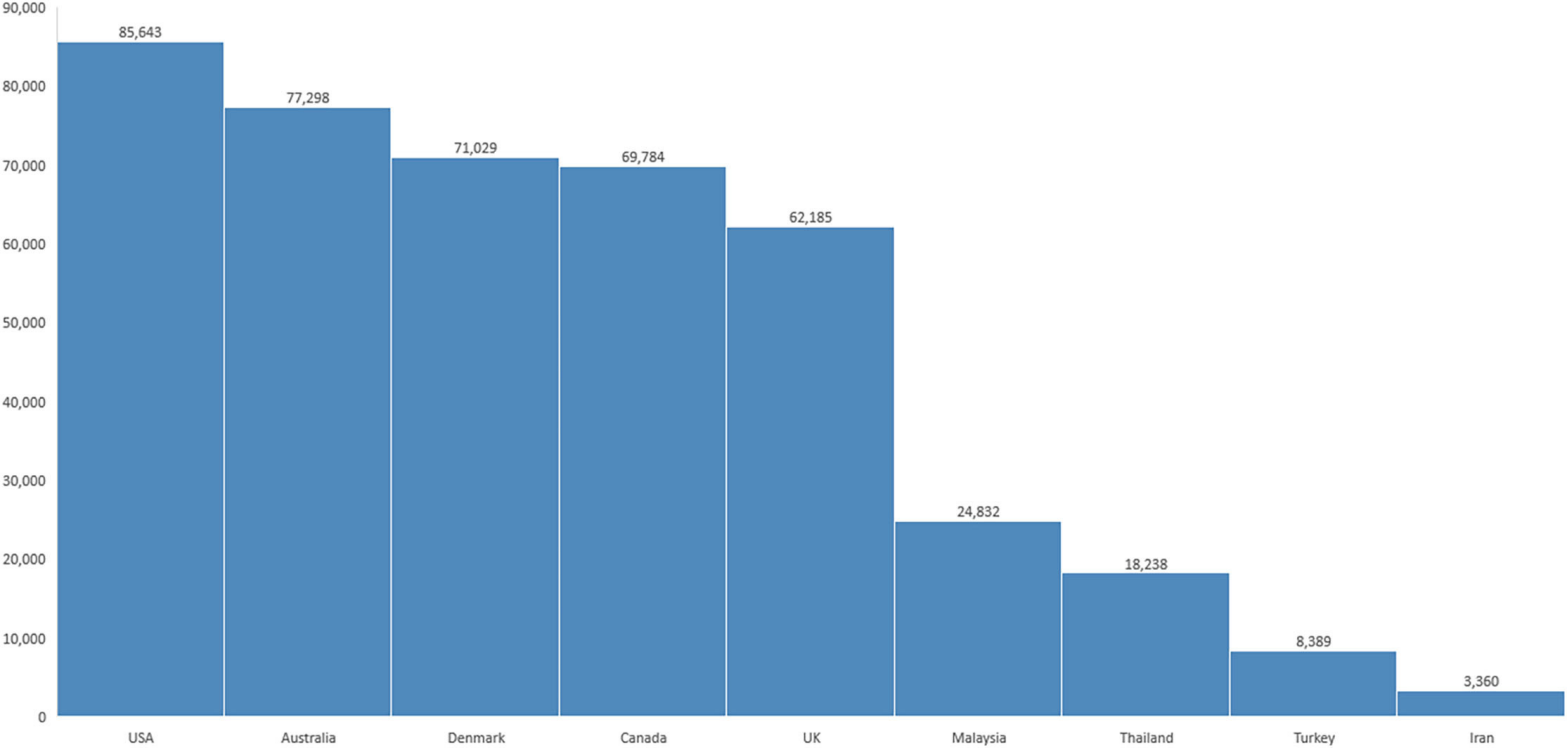
Annual medical resident salaries by country, converted into United States Dollar (10). The mean monthly salary of physician trainees in Iran is 70 million Rials (11). The conversion was made based on the exchange rate to United States Dollar by online currency converter, www.xe.com October 31, 2021 without purchasing power parity.

Heavy debt burdens place financial pressure on residents and this trend might have been accelerated during COVID-19 (9). Figure 1 shows the average salary overall and the average wage and benefits among medical residents in different countries based on the online calculator and up-to-date exchange rate (10). Regardless of residency year and university tuition fee, this rate may vary based on the location, year, as well as the seniority of physician trainees in different cities. Generally, the medical education in Iran is free of charge however the residents have not been fairly compensated when considering the demands made of them. This condition is considerably different from the salaries paid in neighboring or Asian countries of the same socioeconomic status.

#### Long Shift Hours

The recent Medscape survey on 1,509 US medical residents showed that more than half of US residents spend 1–5 nights per month on call, while nearly 92% overall spend <10 nights working (9). Reports in Iran indicate around 15 night-shifts per month during the first-year residency program in some departments (7).

#### Evaluation

Medical residents undergo academic and practical assessment by their education department regularly once a month, and the universities issue permits for their continuation to higher years only after those assessments. However, in 2021, the insistence of the authorities in deputy of education of the Ministry of Health that centralized examinations for medical residents involve in-person examination of the resident collective, even during the fifth peak of the COVID-19 emerging pandemic, requires answers or investigation. In selected countries, nationwide examinations were postponed due to the COVID-19 pandemic (12) and government rushes new doctors into service (13). In 2020, the Iranian government did the same due to the intensive demand for human resources. Yet, in 2021, the deputy of education unexpectedly decided to hold academic assessments for thousands of candidates and physicians that lasted for many hours in a closed space, even though these candidates had already been assessed on a monthly basis by their own universities. The recent decision to reduce the capacity of residency entrance examinations in the current year made a great concern even at the parliament level. In this time, more than 1,200 protesters have requested legal investigation of the performance of the deputy of education in the Ministry of Health. Conflict in management and various internal regulations have caused dissatisfaction (14). For example, annual exams were held for many years at the university level, it became conjoint for a few years, and now it has been centralized at a national level. Implementing this intervention during the pandemic situation calls for investigation and evaluation.

### Crippling Sanctions, Flying Without Wings

Under conditions where healthcare personnel are frustrated and suffer from intensive fatigue, the medical equipment is worn out, and the country faced crippling sanctions which may have significantly contributed to the damage to the healthcare system (15). Sanctions imposed on Iran mean the cash-strapped administration has insufficient reserves to purchase medical equipment and vaccine which may cause the country and global public health into subsequent challenges. However, it is not an unreasonable demand to expect officials to show more responsible attitudes toward healthcare worker in the current fifth and most intense COVID-19 peak, and to anticipate some support for healthcare trainees from the relevant authorities. It is necessary to remain one step ahead of COVID-19, rather than that this pandemic serve as a tool to create dissatisfaction and push healthcare personnel toward protests as the healthcare policymakers should be more realistic. While fighting the pandemic, front line healthcare workers need support and protection instead of frequently working without adequate equipment and remuneration.

### COVID-19 and Higher Burnout Rates

COVID-19 pandemic professed an unprecedented major challenge to the international scientific community (16) that transcends public health. Accumulative stressors during the COVID-19 pandemic may have health implications, with high rates of depression, anxiety, insomnia, and distress in frontline healthcare providers, which require particular attention (17). Studies acknowledged the stressors, burdens, and psychological requirements of the healthcare workers, and the importance of transformation efforts to mitigate these issues confronting those working on the frontlines during the pandemic (18). A survey on 1,420 international physician trainees revealed that exposure to cases with COVID-19 is significantly associated with higher burnout rates (1). Similarly, researchers found a high prevalence of burnout among healthcare providers during the COVID-19 pandemic in Iran. Physician trainees were found to be at a higher risk of developing burnout. Additional research is necessary to elucidate the cause of this, but concerns about heavy workload, changes in duty schedules, as well as having less access to personal protective equipment, may have caused the phenomenon (19). Although the physician trainees may face a burnout during COVID-19 pandemic, the surveys have shown large dissatisfaction among general people about the handling of the COVID-19 pandemic by the former Iranian administration. In a survey conducted by the Iranian State Television, 78% of people gave poor scores to the former administration performance in handling the pandemic and in taking preventive and control measures (20) although the current health administration made a hope and success with mass COVID-19 vaccination strategy.

### Conflict of Interest, a Serious Scourge in the Iran Health System

Conflict of interest still remains a serious challenge in Iran's health system. The following are considered as the underlying causes of conflict of interest in the healthcare system: the structure of the existing financial system fee for service, lack of transparency in the healthcare system, existence of the problem of having two simultaneous jobs dual practice in governmental and private sector, and lack of an integrated information system and electronic healthcare structure (21). Recently, the conflict of interest challenge has attracted the attention of public and media however there is no clear strategy to resolve the potential conflict of interest in the healthcare management structure.

## Recommendations

1. Based on the Accreditation Council for Graduate Medical Education (ACGME) regulations, institutes should supply sleep facilities that are quiet, safe, private, and must be accessible and available for fellows/residents to support safe patient care and education. Moreover, the institution must ensure satisfactory sleep facilities and safe transportation opportunities for fellows/residents who may be too tired to return home safely (22). These recommendations should be applied in Iranian institutes in cooperation with governing bodies.
2. The residency program in the Iranian medical education system is considered to be a student course and therefore the student is given a low salary, bonus, and almost zero profit-sharing. However, it should be noted that physician trainees are medical doctors and Medical Council members who should work 3–5 years in the frontline of hospitals and while at the same time receiving education. By redefining the residency program as a “job” these issues could be resolved (23). In consideration of the protest against the prevailing educational conditions, as well as the mass resignations of physician trainees in some departments, the importance of turning these individual's situation from “student” into “employee” should be considered.
3. The national educational curriculum of the residency program has not been revised for many years (7). Additionally, the ministry's extensive health and medical functions has virtually sidelined education and research that requires a structural reform. To protect the health of the community and that of physician trainees, the curriculum of education should be revised. Furthermore, the extreme authority of the heads of departments has been highlighted as complicating changes in the status of the residency into a safe condition.
4. These policies should be driven from higher authority levels, such as parliament, Medical Council, Ministry of Health, and universities. It is difficult to convey the deprived nature of stakeholders to higher authorities. Whistleblowers and nongovernmental organizations must be able to report directly to law enforcement and regulatory agencies and to competent authorities without risking loss of protection and risk of reprisal.
5. The short term strategies to reduce further challenges are listed below: increasing salaries and wages, amending some post-graduation commitment laws, standardizing the number of night shifts and working hours, coercing the administration to hire medical staff instead of using trainees as low-cost employees, improving and equipping doctor's on-call rooms, providing welfare and livelihood packages with an emphasis on insurance facilities, and eliminating conflicting interests from those who interfere with education policy-making. The list of challenges has been presented in Table 1; these require a specific strategy to resolve.
6. The ACGME-accredited program has recommended that institutes encourage fellows/residents to alert their higher authorities when they are concerned that another trainee or lecturer may be displaying signs of burnout, substance abuse, depression, suicidal ideation, or potential for violence (22). The same strategies should be implemented in most countries with a high rate of burnout of physician trainees.
7. In the context of COVID-19, the best way to combat burnout seems to be precise organization within the hospital and practical training sessions. Effective measures must be taken at the institutional and individual levels (24). However, a national policy in response to burnout of physician trainees is also necessary.
8. Despite the significant successes in the field of knowledge and skills transfer in various clinical areas, insufficient attention is paid to personal development and communication skills. Although attention to medical ethics and professional commitment has been emphasized in the latest version of health education reform in Iran (25), it is questionable to what extent this is planned for implementation and adhered to in practice. In a report by Medscape, 84% of medical residents noted a positive relationship with attending physicians (9) however, there is no nationwide report to show this satisfaction rate among physician trainees in Iran.
9. Despite several reforms within the last few decades, the Iran Ministry of Health, as the steward of the healthcare system in Iran, has not been performing efficiently enough to respond clearly to emerging challenges (26). With 67 medical universities across 31 provinces, Iran is currently among the few countries with medical education integrated with healthcare services. Reform on the medical education seems to be mandatory even if the policy makers reach a decision to constrain the medical education as an independent organization.
10. Protecting healthcare providers is a significant component of public health measures during the COVID-19 pandemic (27). Special interventions to promote mental wellbeing in healthcare workers during COVID-19 need to be implemented immediately.
11. The challenge of ensuring educational equity is remarkable. Different types of quotas and exemptions apply to physician trainees administrative process in Iran. By law, the children of faculty members are allowed to change the majors of their studies (for example from veterinary sciences to pharmacy), or change the city in which they study, easily, while these possibilities are not available to other candidates (28). Recognizing disparities in education opportunities may present better prospects to elites and stakeholders. The governmental body had to become directly engaged in health policy implementation over the past years in Iran (29), without the involvement of non-public sectors and non-governmental organizations. Therefore, policies to control governmental monopoly should be placed.

## Conclusion

With a mixture of success and failure, Iran's healthcare system has undergone several major reforms. Unless this essential transformation takes place within the healthcare system, sustainable development in healthcare may remain a moving goal (29). The performance of healthcare authorities in dealing with physician trainees has not been analyzed. Eliminating potential conflicts of interests in decision-making and administrative process, as well as ensuring sustainable resources, are key elements. Our point is that of unfavorable job description of physician trainees that have been neglected by researchers and decision makers. The lack of health policy in this issue may causes dissatisfaction, outburst, and might affect negatively on the contextual social capital. Healthcare reform will require policy makers to change their attitudes toward young physicians otherwise the country can be expected to have to face a tsunami of migration, brain drain, and lack of human resources in near future.

Healthcare workers are mourning the death of their colleagues and are frustrated and tired, but are still standing tall in the defense of the nation against pandemic. It is imperative that officials make haste in presenting a plan to support healthcare workers, and medical residents in particular. Physician trainees are the foot soldiers and front line of the healthcare system in the battleground for protecting people, and their hardship deserves to be noticed, if the authorities would just take note.

## Author Contributions

All authors listed have made a substantial, direct, and intellectual contribution to the work and approved it for publication.

## Conflict of Interest

The authors declare that the research was conducted in the absence of any commercial or financial relationships that could be construed as a potential conflict of interest.

## Publisher's Note

All claims expressed in this article are solely those of the authors and do not necessarily represent those of their affiliated organizations, or those of the publisher, the editors and the reviewers. Any product that may be evaluated in this article, or claim that may be made by its manufacturer, is not guaranteed or endorsed by the publisher.
